# Structure-function relationships of cholesterol mobilization from the endo-lysosome compartment of NPC1-deficient human cells by β-CD polyrotaxanes

**DOI:** 10.1371/journal.pone.0268613

**Published:** 2022-12-30

**Authors:** Shayak Samaddar, Debosreeta Bose, Bradley P. Loren, Joseph L. Skulsky, Olga Ilnytska, Zachary J. Struzik, Judith Storch, David H. Thompson

**Affiliations:** 1 Department of Chemistry and Purdue Center for Cancer Research, Bindley Bioscience Center, Purdue University, West Lafayette, Indiana, United States of America; 2 Department of Nutritional Sciences and Rutgers Center for Lipid Research, Rutgers University, New Brunswick, New Jersey, United States of America; Azienda Ospedaliero-Universitaria Santa Maria della Misericordia, ITALY

## Abstract

Niemann-Pick Type C is a rare metabolic disorder characterized by the cellular accumulation of cholesterol within endosomal and lysosomal compartments. 2-Hydroxypropyl-β-cyclodextrin (HP-β-CD) containing polyrotaxanes represent an attractive approach for treating this disease due to their ability to circulate in the blood stream for longer periods of time as a prodrug form of HP-β-CD. Once inside the cell, the macromolecular structure is thought to break down into the Pluronic precursor and the active cyclodextrin agent that promotes cholesterol mobilization from the aberrant accumulations within NPC-deficient cells. We now report that both cholesterol and decaarginine (R_10_) endcapped polyrotaxanes are able to remove cholesterol from NPC1 patient fibroblasts. R_10_ endcapped materials enter these cells and are localized within endosomes after 16 h. The cholesterol mobilization from endo-lysosomal compartments of NPC1 cells by the polyrotaxanes was directly related to their extent of endcapping and their threading efficiency. Incorporation of 4-sulfobutylether-β-cyclodextrin (SBE-β-CD) significantly improved cholesterol mobilization due to the improved solubility of the compounds. Additionally, in our efforts to scale-up the synthesis for preclinical studies, we prepared a library of polyrotaxanes using a solid phase synthesis method. These compounds also led to significant cholesterol mobilization from the cells, however, cytotoxicity studies showed that they were substantially more toxic than those prepared by the solvent-assisted method, thus limiting the therapeutic utility of agents prepared by this expedited method. Our findings demonstrate that complete endcapping of the polyrotaxanes and improved solubility are important design features for delivering high copy numbers of therapeutic β-CD to promote enhanced sterol clearance in human NPC1-deficient cells.

## Introduction

Niemann-Pick Type C disease (NPC) is a fatal, neurodegenerative, lysosomal lipid trafficking disorder resulting from aberrant accumulation of unesterified cholesterol and glycosphingolipids in the late endosomal/lysosomal (LE/LY) compartment within cells of many organs including brain, liver, lungs, and spleen [1]. This rare autosomal recessive disease has a broad spectrum of clinical phenotypes, including severe liver disease, breathing difficulties, developmental delay, seizures, decreased muscle tone, lack of coordination, feeding problems, and vertical gaze palsy [2]. Life expectancy for NPC patients is generally below 20 years of age, although later onset cases have also been reported [3]. Its prevalence in Western countries has been reported to be as low as 1:120,000 to 1:150,000 live births [3]. The genetic cause of the disease has been traced to loss of function mutations in the genes encoding for Niemann-Pick Type C1 (NPC1) or Niemann-Pick Type C2 (NPC2) proteins [4, 5]. Most patients (90–95%) diagnosed with NPC have mutations in the NPC1 gene [4]. At present, there is no FDA approved therapy for this rare genetic disorder, making it a serious unmet medical need.

2-Hydroxypropyl-β-cyclodextrin (HP-β-CD) has shown efficacy in reducing aberrantly accumulated cholesterol in both the mouse and cat models of NPC [6, 7]. Dextran conjugates of β-cyclodextrin have also been reported as active toward cholesterol mobilization from the endo-lysosomal compartments of NPC after endocytosis [8]. Weekly *s*.*c*. administration of HP-β-CD in NPC mice reduced cholesterol levels in most organs except lungs and brain [6]. Notably, its poor pharmacokinetic properties due to rapid renal excretion necessitated the use of high doses (4000 mg/kg) administered at frequent intervals to achieve the therapeutic effect [6]. The severity of the disease and the apparent inability of HP-β-CD to cross the blood-brain barrier efficiently has led to clinical trials for HP-β-CD derivatives that employ an intrathecal [9] route of administration. Phase 1 clinical trials with VTS-270 showed encouraging safety and effectiveness data when administered via monthly *i*.*t*. injection at a 900 mg dose. VTS-270 was evaluated in a Phase IIb/III prospective, randomized, double-blind, sham-controlled study to further probe its safety and efficacy via *i*.*t*. injections every 2 weeks at doses of either 900, 1200, or 1800 mg [9]. High doses of HP-β-CD have been reported to cause significant ototoxicity for intrathecal administration [9, 10]. Additionally, significant pulmonary toxicity was observed in the cat model of NPC when the agent was given subcutaneously to achieve hepatic and neurological benefits [11]. Consequently, a safer formulation that improves the pharmacokinetic profile and avoids off-target toxicity of this promising drug candidate is highly desirable.

We have previously reported the synthesis and cholesterol mobilization from endo-lysosomal compartments of NPC1 cells by β-cyclodextrin (β-CD)-based polyrotaxanes (PR) as potential NPC therapeutics [12–15]; findings that were subsequently corroborated using compounds of similar structure [16, 17]. Polyrotaxanes are non-covalent self-assemblies derived from macrocycles that are threaded onto a polymer core followed by the attachment of a bulky molecule (or endcap) to prevent dethreading of the macrocycle. Our efforts have focused on threading various β-CD derivatives onto Pluronic triblock copolymers. The hydrophobic central polypropylene glycol (PPG) block of these copolymers provides a favorable interaction with the hydrophobic central core of the β-CD molecules. The number of β-CD molecules threaded onto the polymer impacts not only the molecular weight of the assembly, but also the rigidity of the PR structure. This, in turn, has an impact on the pharmacokinetics and pharmacodynamics of the PR *in vivo*, with highly threaded variants exhibiting longer blood residence times and depositing primarily in the liver [14]. Variants with lower threading efficiencies, however, are more rapidly cleared through the kidneys [14].

In an effort to more thoroughly explore how PR structure impacts its ability to clear cholesterol from NPC1-deficient cells, we now report the synthesis and testing of two different PR libraries. The first compound collection was synthesized using a previously reported solvent-assisted threading protocol [12], wherein both the polymer core structure and β-CD type were varied. A simplified solvent-free mechanochemical synthesis approach was also developed and used to produce the second compound library. This solid phase method, conceptually related to the method of Tanaka and coworkers [18], was used to fabricate PR containing a nominal 7:3 molar ratio of 2-hydroxypropyl-β-cyclodextrin (HP-β-CD) and 4-sulfobutylether-β-cyclodextrin (SBE-β-CD) with similar polymer cores to evaluate the effect of threading efficiency on the ability of these PR to mobilize cholesterol from the endo-lysosomal compartments of NPC1 patient fibroblasts [19].

## Materials and methods

### Materials

Pluronic triblock copolymers L35 (EO 22, PO 16) and L81 (EO 6, PO 43), HP-β-CD (DS 7–8), methyl-β-cyclodextrin, carbonyldiimidazole (CDI), triethylamine (TEA), and tris(2-aminoethyl)amine (TAEA) were purchased from Sigma-Aldrich. SBE-β-CD (DS 6.5) was a gift from Ligand Pharmaceuticals (San Diego, CA). Toluene, dichloromethane (DCM), and acetonitrile (ACN) were dried using a PPT Solvent Drying Apparatus (Nashua, NH) prior to use. Slide-A-Lyzer™ cellulose dialysis cassettes (2 kDa molecular weight cutoff (MWCO)) were obtained from Thermo Fisher Scientific and immersed in deionized water for at least 30 min prior to use. Ultrapure water (resistivity ≈ 18.2 MΩ/cm^−1^) was generated from a Barnstead MicroPure water purification system. Human fibroblasts from an NPC1 disease patient (GM03123) were obtained from Coriell Institute of Medical Research (Camden, NJ). Filipin III and Heparin sodium salt (from porcine intestinal mucosa) were purchased from Sigma.

### Nuclear magnetic resonance spectroscopy

^1^H NMR spectra were collected using a Bruker AV-III-500-HD spectrometer fitted with a cryoprobe. Spectra were recorded at 25°C at a concentration of approximately 10 mg PR/1 mL in DMSO-*d*_*6*_.

### General procedure for preparation of EDA-modified Pluronics

The Pluronic precursor (5 g) was azeotropically dried four times from toluene and evaporated overnight under a < 100 μm Hg vacuum. The Pluronic was then suspended in dry ACN (95 mL) before addition of CDI (10.67 g) with stirring for 5 h. Water was then added (4 mL) to quench unreacted CDI, followed by addition of ethylenediamine (EDA, 8.8 mL) and stirring at 20°C overnight. Partial solvent removal by rotary evaporation to reduce the volume by approximately two thirds was performed before adding water (37.5 mL) and dialyzing the mixture for 3 d against 30% ethanol in water using 2 kD MWCO dialysis cassettes. The dialysate was then subjected to rotary evaporation to remove as much solvent as possible before azeotropically drying the product three times from toluene.

### General method for decaarginine endcapping of EDA-modified Pluronics (PR1 & PR2)

EDA-modified Pluronic (0.62 g) was suspended in 38 mL hexanes and stirred overnight at 20°C before adding 6.37 g of HP-β-CD. The resulting suspension was bath sonicated for 1 h, probe sonicated for 30 min, and then stirred at 20°C for 3 d. The solvent was removed by rotary evaporation, followed by addition of dry ACN (20 mL) and CDI (67 mg) with stirring of the mixture for 5 h at 20°C under Ar. Solid R_10_ (0.992 g) was added to the reaction mixture with stirring at 20°C under Ar. After stirring for 12 h, triethylamine (0.59 mL) was added and the mixture stirred for an additional 24 h at 20°C under Ar. The mixture was then processed by rotary evaporation of the solvent, dissolution of the solid in ~25 mL deionized water, and dialysis of the aqueous solution against deionized water using 6–8 kD MWCO dialysis cassettes.

### General method for preparation of α,ω-bis-tris(2-aminoethyl)amine-modified Pluronics

The method used for the synthesis of all TAEA modified Pluronics in this study generally followed the approach described by Mondjinou *et al*. [12] Pluronic L81 (3.64 mmol) was azeotropically dried with toluene and placed under a < 100 μm Hg vacuum overnight before dissolving in dry ACN (200 mL) and adding CDI (37.5 mmol). The solution was stirred under an Ar atmosphere overnight. DI water (8 mL) was then added to quench the unreacted CDI and the reactor vented to air to enable CO_2_ gas evolution for 20 min. Tris(2-aminoethyl)amine (181.8 mmol) was then added and the solution stirred at 20°C overnight. Roughly one third of the ACN was removed by rotary evaporation before addition of 30% EtOH and transfer of the solution to 2 kDa MWCO dialysis cassettes. The product was dialyzed over 3 d against 30% EtOH. The solvent was then removed and dried under vacuum to yield α,ω-bis-tris(2-aminoethyl)amine Pluronic intermediate (Pluronic-TAEA) as a viscous liquid.

### Solvent-assisted synthesis of cholesterol-endcapped HP-/SBE-/Me-β-CD polyrotaxanes (PR3 –PR8)

These compounds were prepared as previously described [12, 19]. In brief, the β-CD derivative(s), at a total CD equivalency of one CD unit per two PPG monomer repeats in the Pluronic, was added to a solution of TAEA-Pluronic in hexanes and the slurry briefly bath and probe sonicated before stirring vigorously for 3 d. Cholesteryl chloroformate in DCM was then added and the mixture stirred for 1 d before purification by dialysis (6–8 kDa MWCO membranes) against DMSO for 1 d and deionized H_2_O for 4 d. Lyophilization yielded the product as a white powder. Threading efficiencies were determined by integration of the protons on the cyclodextrin derivatives relative to the CH_3_ group of the PPG block of the Pluronic polymer, wherein 100% threading efficiency occurs at a 2:1 ratio of PPG monomer units:CD. Prior work by Thompson and coworkers has shown that PR prepared in this manner have free CD levels that are ≤5% [12, 13]; HPLC analysis of these samples reveal HP-β-CD levels of <1%. ^1^H NMR (500 MHz, DMSO-d_6_): *δ* = 4.1–5.1 (b, C_1_-H and C_6_-OH of CDs), 3.5–3.8 (m, C_3,5,6_-H of CD), 1.6 (b, (CH_2_)-SO_3_^-^), 1.0 (d, CH_3_ of PPG).

### Mechanochemical synthesis of cholesterol-endcapped HP-/SBE-β-CD polyrotaxanes (PR9 –PR 20)

A mixture of solid TAEA-Pluronic and the desired β-CD variants (500 mg of Pluronic + β-CD mixture at varying ratios) were loaded into agate jars with agate balls and processed in a planetary ball mill (PQN04, Across International) at 350 rpm for varying periods of time (24–96 h). For each sample, the number of 6 mm (30–35) and 10 mm (0–5) balls varied. Endcapping with cholesteryl chloroformate and TEA was performed in DCM as previously described [19]. In all cases, purification was accomplished by multiple ether washes and the product collected by centrifugation, followed by dialysis against deionized H_2_O using 6–8 kDa MWCO dialysis cassettes. Each sample was lyophilized to obtain the PR as a white powder. Threading efficiencies were determined as described above. ^1^H NMR (500 MHz, DMSO-d_6_): *δ* = 4.5–5.1 (b, C_1_-H and C_6_-OH of CD), 3.5–3.8 (m, C_3,5,6_-H of CD), 1.6 (b, (CH_2_)-SO_3_^-^), 1.0 (d, CH_3_ of PPG).

### Evaluation of cholesterol mobilization by β-CD and PR derivatives in NPC1 cells

Cell culture was performed as previously described [12, 13] with minor modification. Briefly, NPC1-deficient cells were seeded in 12 well cell culture plates with cover slips using Eagle’s minimum essential medium with Earle’s salts supplemented with 15% FBS, 1% PenStrep and 1% L-glutamine. Cells were seeded at a density of 2 X 10^4^ cells per well and incubated at 37°C with 5% CO_2_ for 36–48 h until they reached 85–90% confluency. The compounds were solubilized in DMSO (with sonication if needed) and diluted in culture medium to yield a final concentration equivalent to 25 μM β-CD monomer. The final DMSO concentration was ≤ 0.01% v/v. Cells were treated with these solutions for 24 h at 37°C with 5% CO_2_. Following the incubation period, cells were washed with PBS and fixed with 4% paraformaldehyde solution for 30 min. After fixation, cells were washed with chilled PBS and stained twice with 0.05 mg/mL filipin III for 1 h at 37°C, with PBS washing after each staining period. The cover slips with filipin-stained cells were mounted on glass slides with Fluoromount G and sealed with clear nail polish. Images were taken at 40x magnification on an Olympus FSX microscope equipped with a DAPI filter set. Filipin accumulation in cells was determined using NIS-Elements selection tools and calculated as a ratio of filipin stain area to total cell area in individual cells, as previously described [13]. Filipin staining in treated cells were normalized to filipin accumulation in untreated NPC1-deficient fibroblasts and presented as percent cholesterol, with 100% representing untreated, cholesterol laden NPC1 deficient cells. This method typically gave filipin staining values that were ±15% between replicate samples. MTS assays were performed as per the manufacture’s protocol (Promega®) to evaluate the apparent toxicities of the polyrotaxanes at the concentrations used.

### Synthesis of fluorophore-labeled PR derivatives (PR1* and PR2*)

Fluorescently labeled PRs (PR1* and PR2*) were synthesized according to the following procedure. The polyrotaxane (1 equivalent) was placed in a septum sealed 2-neck round bottom flask equipped with a magnetic stir bar. Dry DMF (5 mL) was added under Ar and the mixture stirred before adding CDI (3 equivalents) to the solution from a 9 mg/mL stock solution in dry DMF. This mixture was stirred for 3 h before adding 2,2’-(ethylenedioxy)bis(ethylamine) (3 equivalents) to the reaction from a 2.5% stock solution in dry DMF and stirring for an additional 16 h. Fluorescein isothiocyanate (3 equivalents) was then added to the reaction mixture with stirring for another 12 h. The solution was then diluted with 15 mL of DI water and dialyzed against DI water using 6–8 kDa MWCO regenerated cellulose dialysis membranes over 3 d. The dialyzed polyrotaxane was then centrifuged to remove any water insoluble particles and the final product lyophilized to yield an orange powder (29 mg).

### Localization of fluorescently-labeled PR derivatives

NPC1-deficient cells were grown on glass coverslips. FITC-conjugated PRs were reconstituted in DMSO; the final polymer concentration used for the treatment yielded the equivalent of 25 μM free HP-β-CD. Compounds were added to NPC1-deficient fibroblasts for 1, 2, 6, 16 and 24 h. After incubation, the cells were rinsed with PBS and incubated with 75 nM Lysotracker Red DND 99 (Thermo Fisher Scientific, Waltham, MA) for 45 min in complete medium. Cells were rinsed with PBS, fixed in 4% paraformaldehyde (Electron Microscopy Sci., Halfield, PA) for 10 min, stained with DAPI to visualize nuclei (Thermo Fisher Scientific) and mounted using Fluoromount-G Slide Mounting Medium (Electron Microscopy Sci). Images were acquired using a Zeiss LSM710 confocal microscope (Carl Zeiss Inc., Thornwood, NY) equipped with a 63x, 1.4-numerical-aperture Zeiss Plan Apochromat oil objective.

### FACS analysis for cellular uptake of fluorescently-labeled PR

NPC1-deficient cells were grown in 12 well plates as described above. At 80% confluency, cells were treated with 25 μM HP-β-CD equivalent PR1* and incubated for 2, 8 or 24 h under 37°C, 5% CO_2_ and 95% relative humidity. Additionally, to inhibit heparan sulfate proteoglycans (HSPG) receptor mediated uptake of PR1*, cells were incubated in the presence of 30 μg/mL heparin for 24 h. After incubation, cells were thoroughly rinsed with 1X PBS before trypsinization. Trypsinized cells were resuspended in 1 mL 1X PBS for flow cytometric analysis with BD LSR Fortessa equipped with a 488nm laser to quantify FITC fluorescence. Mean fluorescence from at least 500 cells were quantified. The experiment was performed in triplicate.

## Results and discussion

Two libraries of compounds were prepared for this study, one using hexane-assisted synthesis [20] and the other using a solid state mechanochemical synthesis approach. The materials were characterized by ^1^H NMR to determine the relative ratios of β-CD, threading efficiency, cholesteryl endcapping extent, and molecular weight. The results are summarized in Table 1 (solvent-assisted) and Table 2 (solid state).

**Table 1:** Compositions observed for TAEA-Pluronic polyrotaxane library prepared by hexane solvent-assistance.

	Pluronic core	Ave. #	Ave. #	Ave. #	Total # CD/PR	Endcap	Threading Efficiency (%)	MW (kDa)
		HP-β-CD/PR	SBE-β-CD/PR	Me-β-CD/PR		(# & Type)		
**PR1**	L81	18	0	0	18	2, R_10_	82	31.9
**PR2**	L35	4	0	0	4	2, R_10_	50	11.4
**PR3**	L81	17	0	0	17	4, Chol	75	32.6
**PR4**	L35	6	0	0	6	4, Chol	77	17.3
**PR5**	L35	4	0	0	4	4, Chol	50	9.6
**PR6**	L35	4	2	0	6	4, Chol	75	13.6
**PR7**	L81	0	0	10	10	4, Chol	47	17.7
**PR8**	L81	12	6	0	18	4, Chol	82	34.0

R_10_ = Decaarginine endcaps. Chol = Cholesteryl endcaps.

**Table 2:** Compositions observed for cholesterol-endcapped TAEA-L81 Pluronic polyrotaxane library prepared by mechanochemical synthesis.

	Pluronic Core	Ave. #	Ave. #	Total # CD/PR	Endcap	Threading Efficiency (%)	MW (kDa)
		HP-β-CD/PR	SBE-β-CD/PR		(# & Type)		
**PR9**	L81	7	3	10	1.4, Chol	46	20.1
**PR10**	L81	2	3	5	0.1, Chol	23	12.4
**PR11**	L81	0	4	4	0.44, Chol	18	11.5
**PR12**	L81	6	4	10	1.6, Chol	46	20.7
**PR13**	L81	7	9	16	3.8, Chol	73	32.8
**PR14**	L81	4	3	7	1.6, Chol	30	15.8
**PR15**	L81	2	4	6	0.5, Chol	27	14.4
**PR16**	L81	12	6	18	1.3, Chol	81	33.0
**PR17**	L81	2	3	5	0.3, Chol	23	12.4
**PR18**	L81	6	4	10	0.8, Chol	46	20.2
**PR19**	L81	5	3	8	0.6, Chol	36	16.8
**PR20**	L81	3	4	7	0.9, Chol	32	15.9

### Cholesterol mobilization from endo-lysosomal compartments of NPC1 cells

We have previously shown that PR with cholesterol endcaps recruit a variety of serum proteins, particularly apolipoproteins A-I, A-II, A-IV, C-I, C-II, C-III, and E lipoproteins [14], regardless of the cyclodextrin used to prepare the PR. SBE-β-CD was included in the PR syntheses to help improve the solubility of the final PR since earlier work showed that PR without charged CD units often aggregate and are poorly soluble in aqueous solution. The cholesterol endcapped polyrotaxane derivatives were designed with this association in mind to serve as a potential Trojan Horse strategy by promoting their delivery to the LE/LY of NPC cells via LDL receptor mediated endocytosis. Based on our previous studies, we decided to compare the cholesterol mobilization properties of these novel constructs at a cyclodextrin concentration of 25 μM [12, 13, 15, 19]. Satisfyingly, we found that incubation with these PR compounds led to cholesterol clearance from the LE/LY compartment, presumably via receptor mediated endocytosis since a fluorescent analogue of these compounds was shown to colocalize with Lysotracker [15]. It is also possible that bulk phase endocytosis may also be contributing to some of the PR delivery observed [20]; both receptor mediated and bulk phase endocytosis would deliver the agents to the endolysosomal vesicle trafficking system.

**Fig 1:**
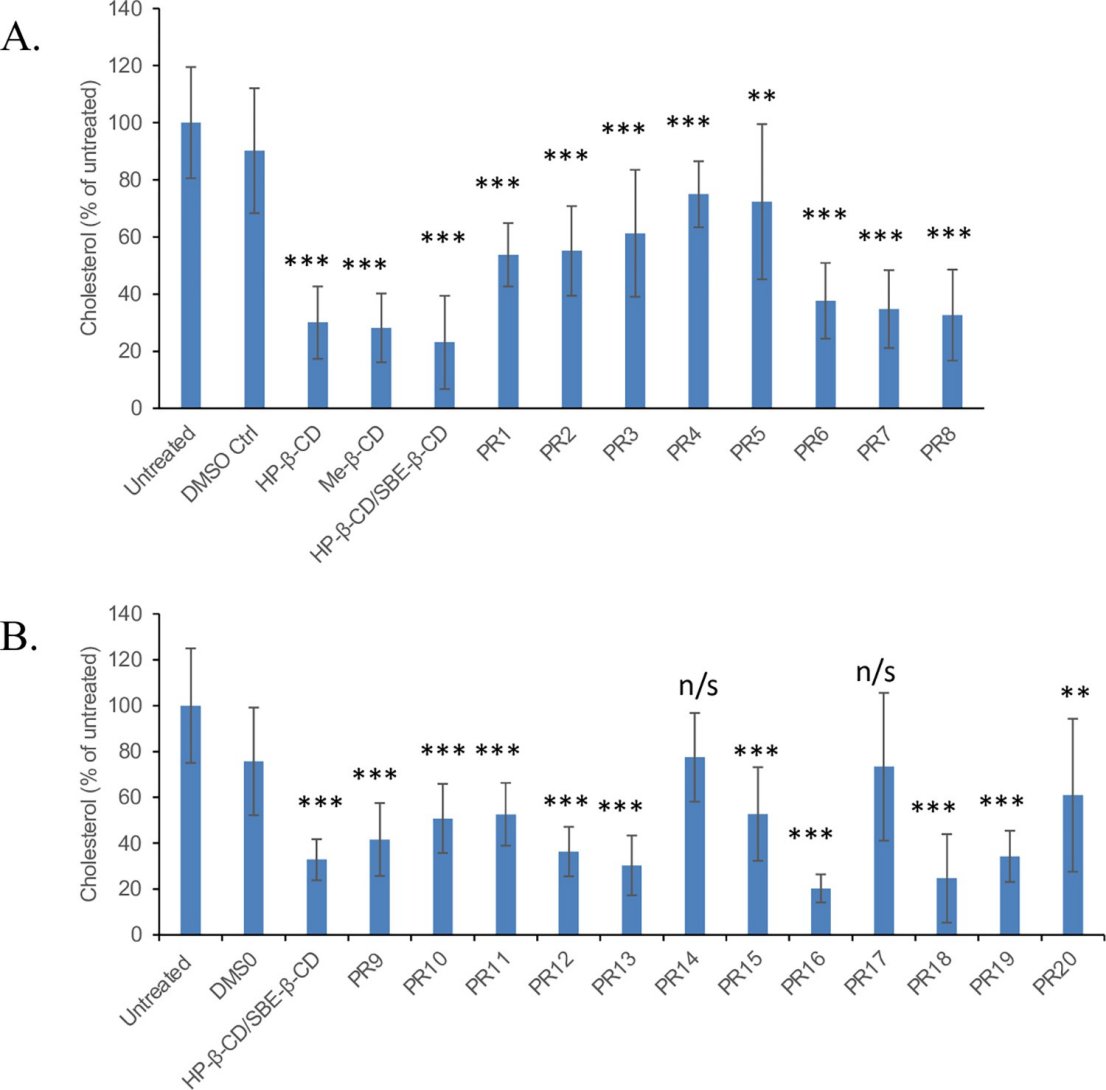
Cholesterol accumulation in NPC1-deficient cells as determined by filipin III staining after exposure to polyrotaxanes for 24 h at 25 μM equivalent β-CD monomer concentration. **A.** Controls and polyrotaxanes **PR1** –**PR8** prepared by the solvent-assisted method. **B.** Controls and polyrotaxanes **PR9** –**PR20** prepared by solid state mechanochemical synthesis. Data are expressed relative to DMSO-treated controls and are an average +/- S.D. of two separate experiments with 30 cells imaged per condition in each experiment. (t-test vs. DMSO control, **p<0.01; ***p<0.001).

Cell-penetrating peptides are known to efficiently transport a variety of molecular and macromolecular cargo across plasma membranes [21, 22] via energy-dependent and/or energy-independent pathways depending on the nature of the construct [23]. Additionally, multiple studies have utilized such peptides for cellular delivery of therapeutic payloads without any signs of toxicity up to a R_10_ concentration of 60 μM [24–27]. We therefore sought to evaluate the effect of a positively charged R_10_ PR endcap on cholesterol mobilization from the endo-lysosome compartment. Multiple studies have suggested that R_10_ sequences do not cross the plasma membrane of live cells directly (i.e., by electrostatic interaction with negatively charged lipid of cell membrane); rather, they bind to heparan sulfate proteoglycans (HSPG) present on the cell surface, resulting in heparan sulfate-mediated endocytosis [27–31]. Once inside this compartment, HSPG is cleaved by heparinase into oligosaccharide fragments. In the event that the R_10_ endcapped PR materials follow this pathway, these constructs would be released into the cytosol. Although R_10_ constructs can also escape into cytoplasm by endosomal leakage, this phenomenon has not been reported at the low micromolar concentrations used in our study [23]. Under the conditions employed in our experiments, the PR containing endosomes are likely to mature into lysosomes, where the carbamate bonds linking the R_10_ endcaps to the PR core are enzymatically cleaved to enable HP-β-CD dethreading [13]. Once the HP-β-CD units have been dethreaded, they are then available to mobilize aberrantly accumulated cholesterol. To monitor cholesterol mobilization from the endo-lysosomal compartment, we stained treated and untreated NPC1 cells with cholesterol binding fluorescent dye, Filipin III, which has been extensively used to monitor efficiency of NPC therapeutics *in vitro* [32–36]. Fluorescence of treated cells were normalized to untreated controls as 100% cellular cholesterol. Our Filipin III staining data suggest that **PR1** and **PR2**, HP-β-CD polyrotaxanes with R_10_ endcaps, led to cholesterol mobilization from cells, reducing cellular cholesterol by 35–40% relative to untreated controls (Fig 1A). LDL receptor mediated endocytosis of LDL occurs predominantly by caveolar uptake [37], whereas HSPG mediated endocytosis is a clathrin- and caveolin-independent, but dynamin- and flotillin-dependent, pathway [38]. Since both L81- and L35-based polyrotaxanes resulted in similar internalization kinetics and localizations, these findings reveal that cellular uptake is independent of the polymer backbone length. Interestingly, **PR3** and **PR4**, species having similar HP-β-CD content, but with cholesterol endcaps (inferred to be internalized via LDL receptor mediated pathways based on high levels of ApoA and ApoC association [14]) were less effective than the R_10_-endcapped **PR1** and **PR2**.

Inclusion of SBE-β-CD in the HP-β-CD polyrotaxane formulation was intended to boost the PR solubility in physiological saline solutions [19, 39]. Cholesterol mobilization improved when SBE-β-CD was included in the polyrotaxane scaffold. Our results show that **PR6** and **PR8** produced cholesterol mobilization levels that were almost as effective as the monomeric β-CD controls. Direct comparison of **PR4** (6 HP-β-CD) and **PR6** (4 HP-β-CD and 2 SBE-β-CD), both having the same total number of β-CD threaded onto a Pluronic L35 core, shows that inclusion of SBE-β-CD boosted sterol clearance from the endo-lysosomal compartment by approximately 60% of control, while the compound with HP-β-CD alone led to mobilization by only about 25%. The same trend is apparent for PR3 and PR8 with Pluronic L81 cores, where threading of HP-β-CD along with SBE-β-CD lowers the accumulated cholesterol by 70% compared to untreated NPC1 deficient cells, while the compound with HP-β-CD alone reduced sterol by 40%. We infer from these findings that, as intended, the solubility enhancement provided by inclusion of SBE-β-CD units can substantially improve the performance of the polyrotaxanes [40, 41]. Interestingly, the length of the polymer chain and number of CDs threaded on it did not result in a significant difference in cholesterol mobilization effectiveness, as had been expected. For example, comparison between **PR3** and **PR4**, or between **PR1** and **PR2**, two groups of compounds with the same endcaps and Pluronic cores and 3-fold differences in β-CD units per molecule, similar cholesterol mobilization levels were found. Our observations suggest that solubility of the PRs is a critical determinant of therapeutic outcome at a fixed dose of CD units.

**Fig 2:**
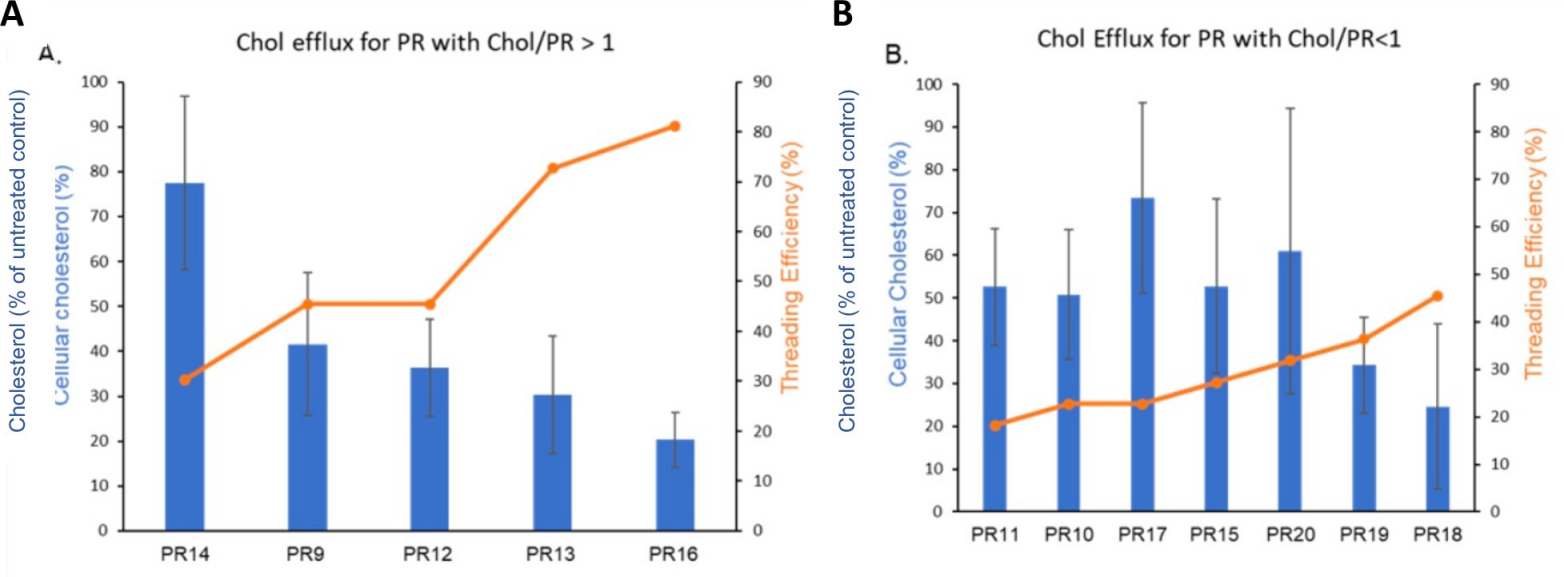
Cholesterol mobilization as a function of polyrotaxane threading efficiency in NPC1 deficient cells. **A.** Cellular cholesterol content as a function of polyrotaxanes with Chol/PR > 1. **B.** Cellular cholesterol content as a function of polyrotaxanes with Chol/PR < 1. Endo-lysosomal cholesterol staining is shown relative to untreated cells (100%).

We also evaluated the cholesterol mobilization efficacy of the materials made via solid state mechanochemical synthesis (Fig 1B). Since previous results suggested L81 as the best Pluronic core for cholesterol mobilization activity and long circulation *in vivo*, we utilized this polymer core in these preparations [14]. Additionally, we also incorporated SBE-β-CD in all the PRs of mechanochemical synthesis as the PRs made in the hexane phase demonstrated the therapeutic benefit of its inclusion. Highly threaded PR such as **PR13** (7 HP-β-CD, 9 SBE-β-CD) and **PR16** (12 HP-β-CD, 6 SBE-β-CD), with threading efficiencies of 73% and 81%, showed the greatest ability to lower cholesterol accumulation, with reductions of approximately 70% and 80%, respectively, compared to untreated NPC1 cells. **PR9** and **PR12,** which had only 46% threading efficiency, reduced endo-lysosomal cholesterol by approximately 40% relative to untreated controls. **PR18,** with a similar CD loading and threading efficiency, showed somewhat increased effectiveness in cholesterol clearance; this may be attributed to the lower cholesterol endcap content in this PR, thereby enabling the β-CD rings to dethread more easily once inside the LE/LY compartment. It important to mention here that extracellular dethreading of PRs is highly unlikely *in vitro* due to serum protein association with the cholesterol endcaps that will sterically block CD dethreading until the carbamate linkage is enzymatically cleaved within the endosome [14]. It should be noted that the higher threading efficiency would lead to a more rigid rod like structure compared to PR with low threading efficiencies that have greater flexibility. Previously, we have analyzed in depth the effect of PR rigidity on the pharmacokinetics of PR in mice. These studies showed that rigid PR circulate longer and preferentially accumulate in liver [14] relative to their more flexible congeners. The data in Table 1 suggests that PR rigidity is linked with increased cholesterol mobilization from the endo-lysosomal compartment. This is consistent with the recent finding that high aspect ratio rod-like particles have an enhanced ability to enter cells [42]. Consistent with this interpretation, PR having threading efficiencies below 32% (e.g., **PR10**, **PR11**, **PR14**, **PR15**, **PR17**, and **PR20**) were not as effective in cholesterol clearance, reducing sterol accumulation by 50% or less relative to untreated cells (Fig 2).

We further explored the role of endcapping extent in the PR library to evaluate the impact of dethreading on cholesterol clearance efficiency. Our findings reveal some important trends that can guide subsequent polyrotaxane designs. First, at a Chol/PR ratio of >1, it can be clearly seen that the cellular cholesterol percentage decreases (i.e., efficacy increases) as the threading efficiency increases (R^2^ = 0.87). Second, for polyrotaxanes with low end capping extent (Chol/PR ratio of <1), no trends in polyrotaxane composition were evident (R^2^ = 0.16). Third, as was observed for the polyrotaxane materials derived from solvent-assisted synthesis, more extensive cholesterol endcapping is essential for producing highly threaded PRs.

### Cellular localization of R_10_-endcapped PR by confocal microscopy

**Fig 3:**
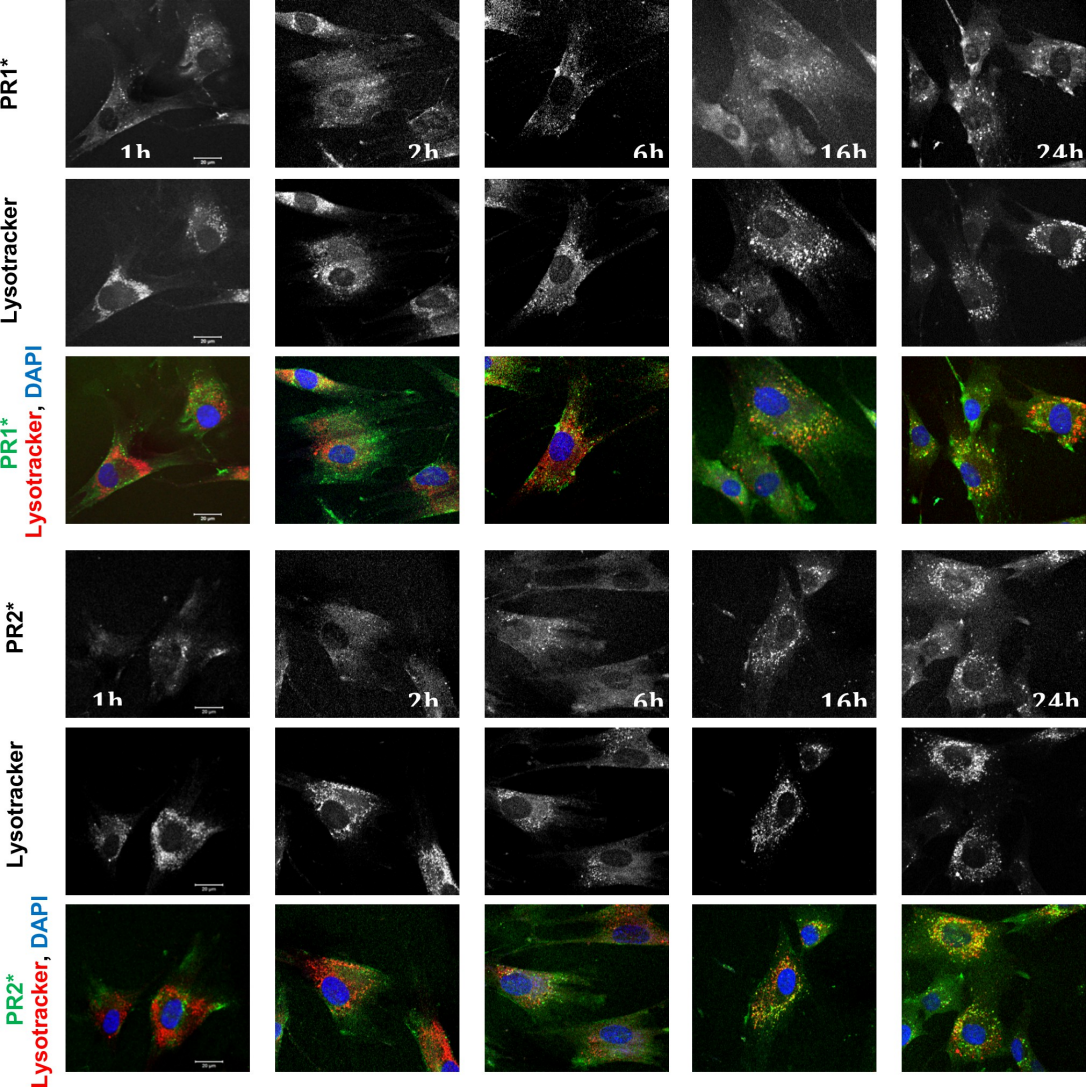
Time course of FITC-conjugated PRs (PR1* and PR2*) localization in NPC1-deficient fibroblasts. Cells were incubated with the compounds for the indicated times (h) and then washed and stained with lysotracker for an additional 45 min. The cells were fixed with paraformaldehyde, followed by staining of nuclei by DAPI. Cells were imaged by confocal microscopy as described in Methods.

The effectiveness of the PR compounds toward cholesterol mobilization likely requires their localization within the lysosomal compartment where cholesterol accumulates. To directly examine the subcellular localization of these compounds, two fluorescent-tagged PR (PR1* and PR2*) were generated as described above. Fluorescence microscopic imaging demonstrated that partial co-localization of HP-β-CD with a lysosomal marker was detected at 6h, and more prominent co-localization was detected after 16 h treatment, indicating that over time the fluorescent PR compounds accumulate in the lysosomes (Fig 3). These data suggest that the R_10_ endcapped PR were efficiently endocytosed, possibly via a receptor mediated endocytosis, and were thus able to mobilize cholesterol from their late endosomal/lysosomal sites of accumulation.

### FACS analysis to evaluate cellular uptake of fluorescently-labeled PR

**Fig 4:**
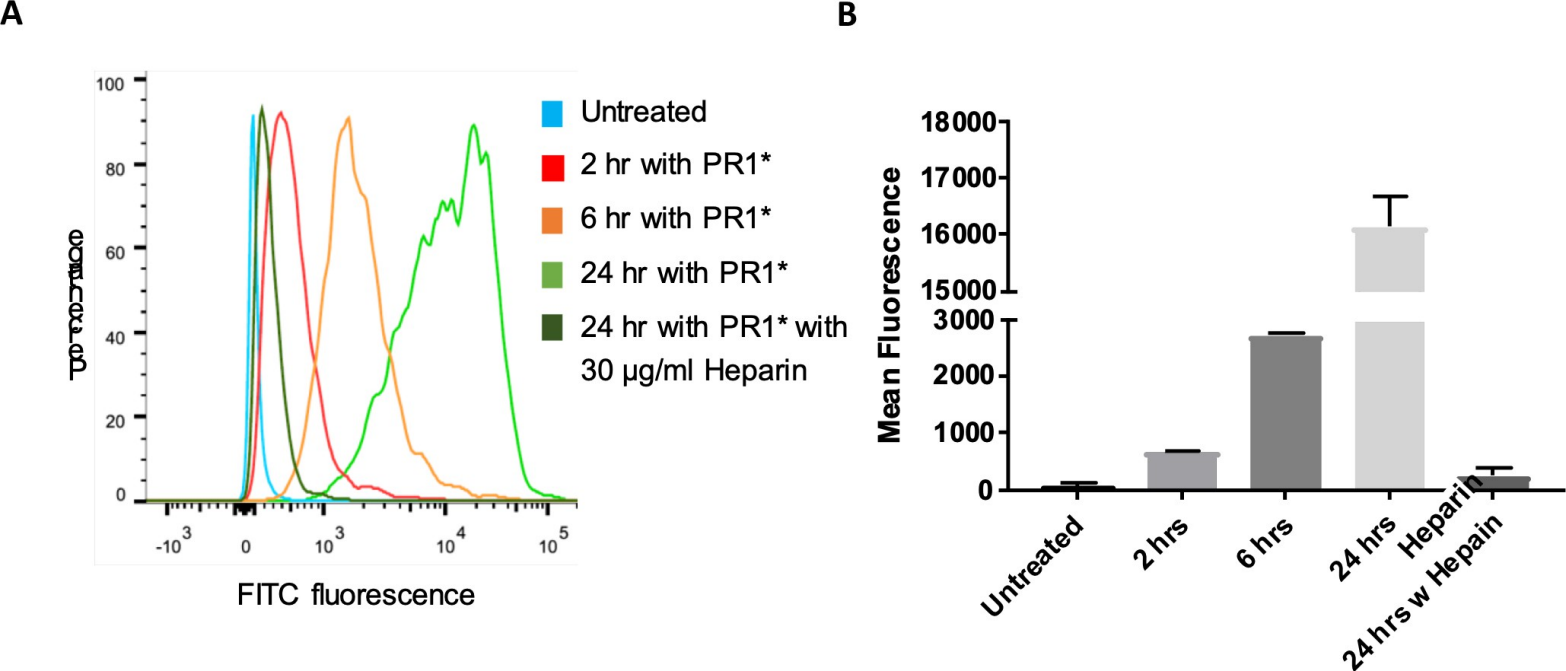
**A.** Histogram plot for FITC fluorescence distribution following PR1* uptake within NPC1 deficient fibroblast cells. **B.** Cellular internalization of PR1* in the absence (2, 6, and 24 h) and presence (24 h) of 30 μg/mL of heparin. Cells were treated for 24 h at 25 μM equivalent β-CD monomer concentration and analyzed by flow cytometry as described in the methods.

Flow cytometry analysis of NPC1 deficient cells with FITC-labelled PR (PR1*) shows enhanced cell-associated FITC fluorescence, consistent with our confocal data showing that internalization of R_10_ end-capped PRs occurs within 2 h of incubation (Fig 4). The cellular fluorescence is maximum after 24 h of incubation with PR1*. Mani *et al*. reported the presence of HSPG receptors in NPC1 fibroblast cells [43]. To probe whether the R_10_ end-capped PRs utilize the HSPG receptor for cellular internalization, we antagonized the HSPG receptor with 30 μg/mL heparin and monitored its effect on PR1* uptake [44–46]. Mean FITC fluorescence of the NPC1 cells show that after 24 h of incubation, internalization of PR1* was ~60 fold higher as compared to internalization of the R_10_ end-capped PR in the presence of heparin. These findings suggest that the R_10_ endcapped PRs are most likely internalized via an energy dependent HSPG pathway.

### Cytotoxicity evaluation PR materials

**Fig 5:**
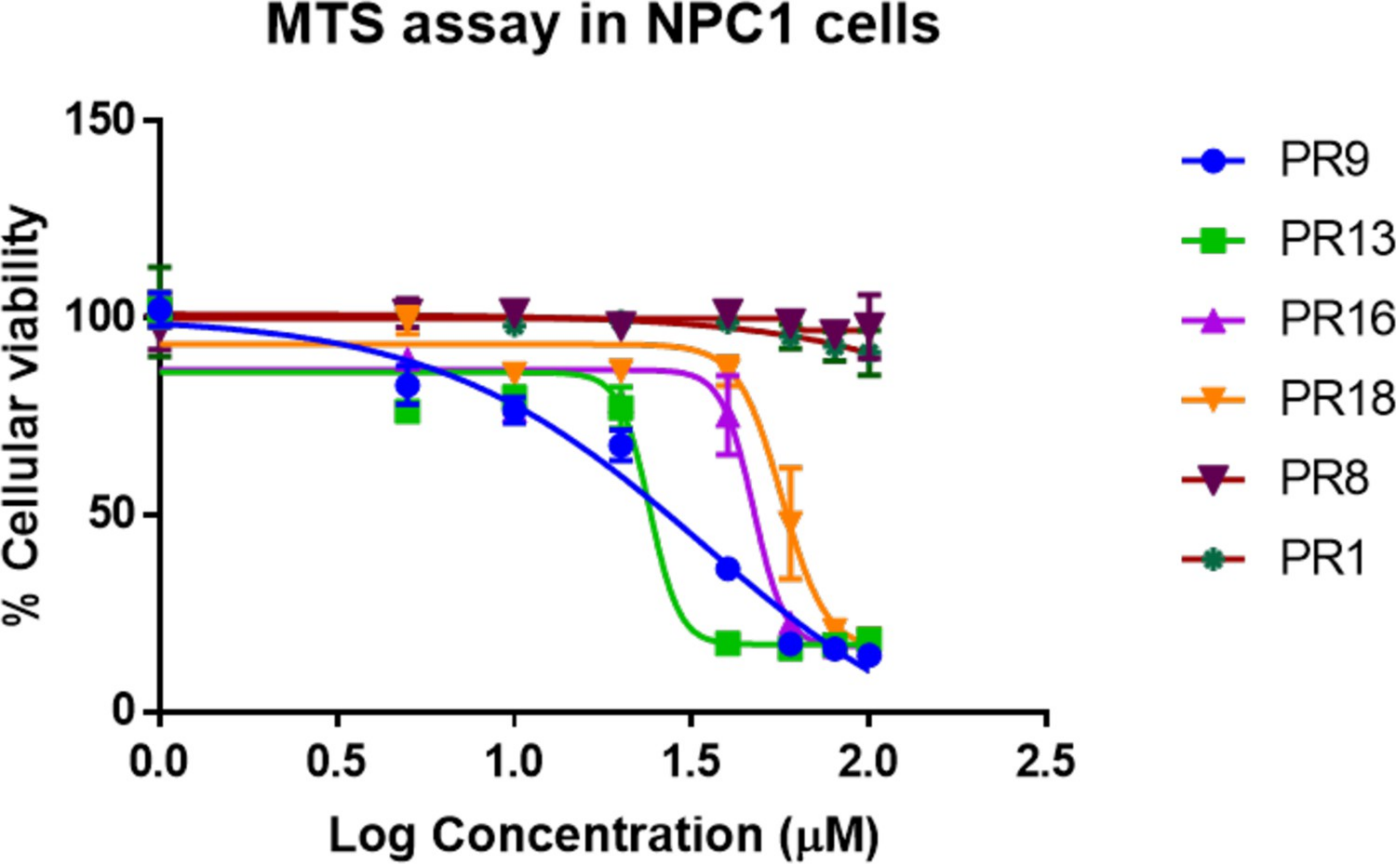
Cytotoxicity analysis of compounds prepared by solid phase synthesis vs solvent-assisted synthesis. NPC1-deficient cells (1500 cells/well) were plated in a 96 well plate for 36 h before treatment with varying concentrations of PRs. Cells were incubated for a further 24 h before measuring toxicity of the compounds by MTS assay according to manufacturer’s protocol.

The clinical success of any drug delivery strategy depends on the ability of the agent to elicit therapeutic benefit without significant toxic effects. In fact, our design approach was devised to use only precursors found in FDA approved materials to obtain the final PR products that would have an “FDA-friendly” toxicity profile. Our cholesterol mobilization studies were based on our previous experience that PRs were efficient at normalizing cellular cholesterol level at a 25 μM cyclodextrin equivalent dose. However, evidence of cell detachment and loss (treated with PRs synthesized by solid phase protocol) during the staining procedure prompted us to compare the toxicity profile of PRs synthesized by the two different methods. Here, **PR9**, **PR13**, **PR16** and **PR18** were used as representative of materials prepared by the solid phase method. Additionally, to compare the effect of end-capping on the toxicity profile, we used **PR1** (R_10_ endcapped) and **PR8** (cholesterol endcapped) as representatives from our library synthesized by solvent-assisted method. Our cytotoxicity evaluations revealed that the materials synthesized by the solvent-assisted method were much less cytotoxic than the compounds prepared by solid phase synthesis. As shown in Fig 5, MTS assay results revealed that the solvent-assisted materials **PR1** (R_10_ eq. concentration of 2.7 μM) and **PR8** did not display significant cytotoxicity at the concentrations tested. By contrast, over this same concentration range, the enhanced toxicity of the compounds prepared by solid phase synthesis was clearly evident. At a concentration of 100 μM, compounds prepared by solid phase synthesis (**PR9**, **PR13**, **PR16**, **PR18**) were significantly more toxic than those prepared by the solvent-assisted method (**PR1** and **PR8**). Further analysis using GraphPad Prism revealed that **PR9**, **PR13**, **PR16** and **PR18** had IC_50_ values of 34 μM, 24 μM, 48 μM and 56 μM, respectively, which severely limits their therapeutic potential. Since both synthesis methods did not contain materials that could contribute to their toxicity and were analytically similar by ^1^H NMR, we attribute the toxicity to a side reaction of the ball milling process that may produce a difficult-to-detect crosslinked PR structures that are cytotoxic. Additional studies to understand the source of this toxicity are needed before further pursuing the solid phase synthesis approach for further biological experimentation.

## Conclusions

In conclusion, we demostrate that polyrotaxnes prepared by solvent-assisted synthesis are capable of mobilizing trapped cholesterol from the late endosomal/lysosomal compartments of Niemann-Pick Type C1 deficient cells. We infer from our findings that the use of R_10_ endcaps helped promote PR internalization via a HSPG mediated pathway such that they become localized in late endosomes/lysosomes where they promote cholesterol egress. Cholesterol mobilization from the endo-lysosome compartments of NPC1 cells is shown to be dependent on the threading efficiency and solubility of the compounds. Rigid rod-like materials with a high number of threaded CDs show better cholesterol mobilization than those with lower CD content. Incorporation of SBE-β-CD to enhance the solubility of the compounds also improves the performance of the PR toward cholesterol mobilization. Although solid phase synthesis is an attractive approach for scalable production of polyrotaxanes, it likely results in crosslinked cyclodextrin polymers that are more toxic than their polyrotaxane counterparts. Challenges associated with mechanochemical synthesis will need to be addressed before this method can be used to produce material for biological experiments.

## Supporting information

S1 File Effect of end-capping, threading efficiency and solubility on cholesterol mobilization from the endo-lysosomal compartments of NPC1-deficient cells. (DOCX)
