# Supplementary material for: Structure-function relationships of cholesterol mobilization from the endo-lysosome compartment of NPC1-deficient human cells by β-CD polyrotaxanes
Source: PLoS One. 2022 Dec 30;17(12):e0268613. doi: 10.1371/journal.pone.0268613 (PMC9803220; doi:10.1371/journal.pone.0268613)
Supplement: S1 File — (DOCX) [file pone.0268613.s001.docx]

**
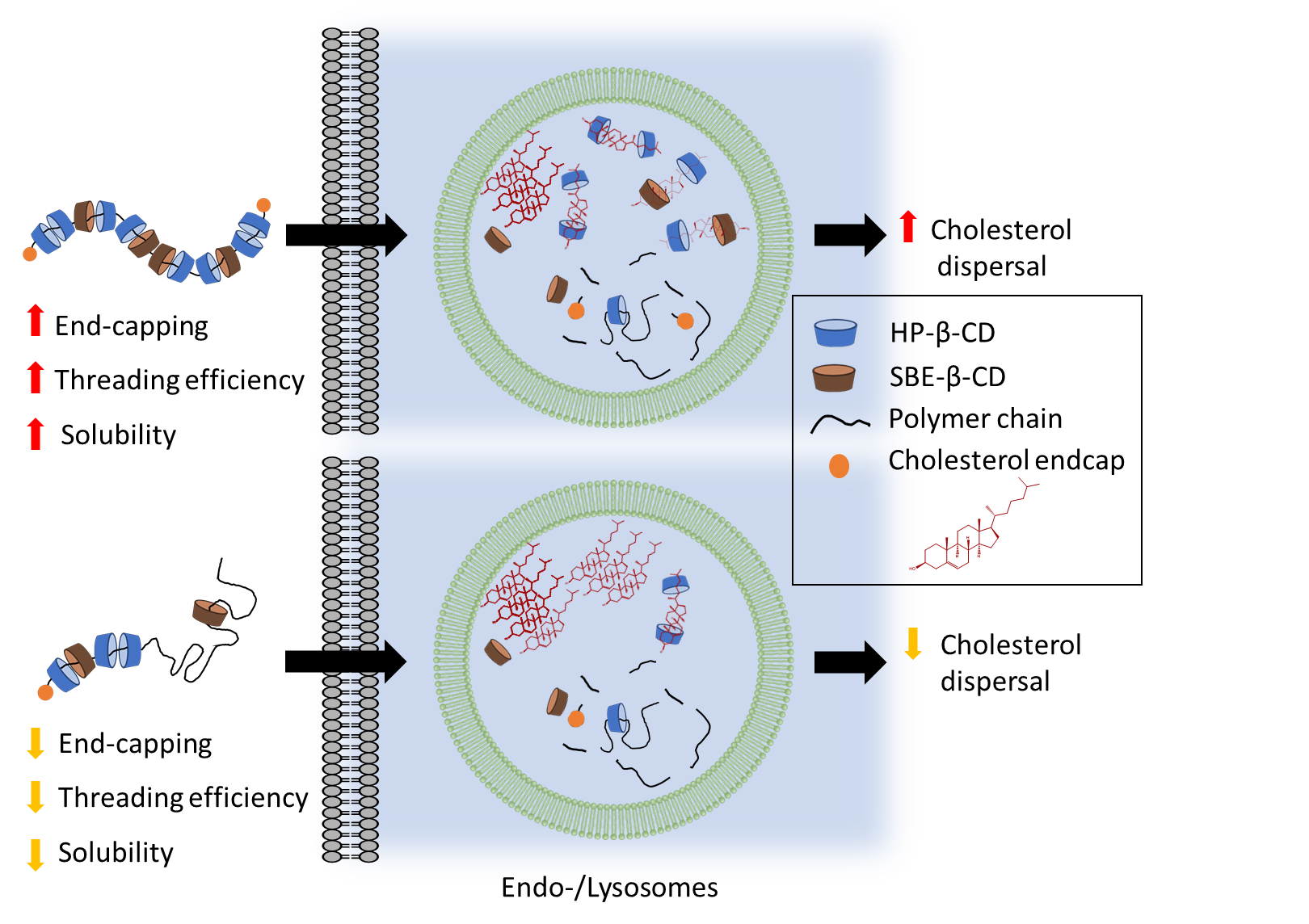
** **Table of content:** Effect of end-capping, threading efficiency and solubility on cholesterol mobilization from the endo-lysosomal compartments of NPC1-deficient cells.
